# P2IFormer: A Multi-Granularity Patch-to-Image Embedding Model for Fault Diagnosis of High-Speed Train Axle-Box Bearings

**DOI:** 10.3390/s25165138

**Published:** 2025-08-19

**Authors:** Weigang Ma, Chaohui Zhang, Ling Chen, Zhoukai Wang, Xing Fan, Yingan Cui

**Affiliations:** School of Computer Science and Engineering, Xi’an University of Technology, Xi’an 710048, China; 2241221110@stu.xaut.edu.cn (L.C.); zkwang@xaut.edu.cn (Z.W.); xing.fan@xaut.edu.cn (X.F.); cuiyan@xaut.edu.cn (Y.C.)

**Keywords:** high-speed train, axle-box bearing, fault diagnosis, time series to image, gramian angular field

## Abstract

The axle-box bearing is a critical load-bearing component in high-speed trains and is prone to failure under long-term heavy-duty operation, affecting both operational efficiency and safety. Current deep-learning-based fault diagnosis methods face two key challenges: difficulty in capturing temporal features across multiple scales simultaneously, and limited capability in modeling local sequential patterns. To address these issues, we propose P2IFormer, a fault diagnosis model based on multi-granularity patch-to-image embedding. The raw vibration sequence is divided into equal-length patch sequences under multiple granularities, each defined by a fixed window size. Each patch is then transformed into a Gramian Angular Field (GAF) image to extract spatial features and generate granularity-specific embedding. A multi-granularity self-attention mechanism is used to model both intra- and inter-granularity dependencies. The resulting multi-granularity features are fused and fed into a softmax classifier for final fault prediction. Experiments conducted under four constant-speed conditions and one variable-speed condition demonstrate that P2IFormer achieves over 99.5% accuracy across all scenarios, significantly outperforming existing CNN- and Transformer-based methods.

## 1. Introduction

**Figure 1:**
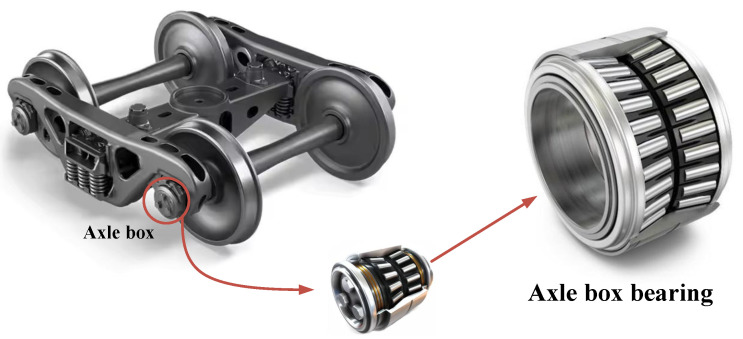
The structural location of the axle-box bearing in a high-speed train bogie.

High-speed trains, as a vital component of modern transportation systems, are widely used for intercity passenger and freight transport due to their high speed and large carrying capacity. The axle-box bearing, shown in Figure 1, a key load-bearing component connecting the wheelset and the bogie, plays a crucial role in ensuring the train’s operational performance and safety. However, due to frequent dynamic loads and complex wheel–rail interactions, axle-box bearings are prone to wear and degradation. If their condition cannot be accurately diagnosed in a timely manner and corresponding maintenance is not carried out, such failures may lead to severe malfunctions or even major accidents. Therefore, developing effective fault diagnosis methods for axle-box bearings is of great practical importance for ensuring the safe operation of high-speed trains, reducing maintenance costs, and improving overall operational efficiency [1,2].

The health condition of high-speed train axle-box bearings is directly related to the safety and reliability of train operations. However, fault diagnosis in practical scenarios faces multiple challenges. On the one hand, it is extremely difficult to obtain sufficient fault samples under real operating conditions and the available data are often highly imbalanced. On the other hand, the complex and variable operating conditions of trains—such as changes in rotational speed and noise interference—significantly increase the difficulty of diagnosis [3,4]. Traditional diagnostic methods typically rely on signal processing in the time domain, frequency domain, or time-frequency domain, combined with machine learning models such as support vector machines for fault identification [5,6,7]. These approaches are heavily dependent on manual feature extraction, which is not only time-consuming and expertise-intensive but also poorly adaptable to variable operating conditions, thereby limiting the accuracy and robustness of diagnostic models [8].

In recent years, the rapid development of deep learning has driven its widespread application in fault diagnosis. Compared with traditional methods, deep learning models can directly extract high-level features from raw vibration signals, reducing reliance on manual feature engineering and significantly improving diagnostic accuracy [9]. Chen et al. [10] proposed a feature-aligned multi-scale convolutional neural network (MSCNN-FA) that converts vibration signals into time-frequency images using short-time Fourier transform (STFT) and incorporates a feature alignment module with a multi-scale convolution strategy, achieving a diagnostic accuracy of 99.2%. Sun et al. [11] developed a fault diagnosis method that combines multi-scale CNN and long short-term memory (LSTM) networks, where the multi-scale CNN extracts local features from time-frequency images and the LSTM captures long-term temporal dependencies, resulting in an accuracy of approximately 98%. Zhao et al. [12] introduced a multi-scale convolutional neural network–bidirectional LSTM–attention mechanism (MSCNN-BiLSTM-AM) approach, in which the multi-scale CNN extracts spatiotemporal features, the BiLSTM captures bidirectional temporal dependencies, and the attention mechanism enhances the representation of key features, achieving a diagnostic accuracy of 99.5%. To address the issue of data imbalance, Hou et al. [13] and Luo et al. [14] designed improved generative adversarial networks (GANs), which achieved diagnostic accuracies of 97.8% and 98.2%, respectively, on the CWRU dataset, demonstrating strong robustness under imbalanced data conditions.

In fault diagnosis research, a widely used approach is to transform time-series signals into two-dimensional representations to enhance the expressive capacity of temporal features and to apply two-dimensional convolutional neural networks (2D-CNNs) for fault identification [15]. Among such methods, the transformation of time-series data into images using the Gramian Angular Field (GAF) has been extensively adopted [16,17]. For example, Bai et al. [18] proposed a GAF-based SEDenseNet method that converts vibration signals into two-dimensional images via GAF and incorporates a squeeze-and-excitation (SE) module to optimize feature extraction, achieving a diagnostic accuracy of 97.5%. Yu et al. [19] presented a GAF-based bearing fault diagnosis approach that similarly maps vibration signals to images and employs a generative adversarial network (GAN) to synthesize training samples, reaching 96.8% accuracy and demonstrating robustness under class-imbalanced conditions. Tong et al. [20] developed a method using the Gramian Angular Difference Field (GADF) combined with channel attention and SimAM attention mechanisms, obtaining 98.7% accuracy. However, the inherently local receptive field of convolutional neural networks limits their capacity to model global contextual information, which can reduce diagnostic performance when handling long sequences or complex fault patterns.

Transformer architectures, through their intrinsic self-attention mechanisms, enable the direct modeling of long-range dependencies across all positions in sequential data, thereby demonstrating substantial efficacy in fault diagnosis [21,22]. For instance, Hou et al. [23] proposed the Diagnosisformer model, which extracts frequency-domain features using Fast Fourier Transform (FFT) and integrates a multi-feature parallel fusion encoder and a cross-flip decoder, achieving a diagnostic accuracy of 99.85% on the CWRU dataset. However, Transformers have certain limitations in modeling local patterns and extracting multi-granularity temporal features. To address these issues, hybrid CNN–Transformer architectures have been proposed. Chen et al. [24] introduced an efficient cross-space multi-scale CNN–Transformer parallel architecture (ECMCTP) that generates time-frequency images using the continuous wavelet transform (CWT) and employs parallel CNN and Transformer branches to capture local and global features, delivering excellent diagnostic accuracy and noise robustness. Han et al. [25] developed a multi-task MT-ConvFormer model that generates time-frequency representations via short-time Fourier transform (STFT) and integrates CNNs’ local feature extraction with Transformers’ long-range dependency modeling, achieving a diagnostic accuracy close to 98%.

To address the limitations of existing fault diagnosis methods in local feature modeling and multi-scale feature extraction, this paper proposes P2IFormer, a fault diagnosis model for high-speed train axle-box bearings based on multi-granularity patch-to-image embedding. The proposed approach first segments raw vibration signals into equal-length patch sequences at multiple granularities using a set of predefined window sizes, enabling structured multi-granularity modeling of the time series. For each granularity level, the individual patches are then transformed into corresponding Gramian Angular Field (GAF) images, enhancing the visual expression of local temporal features. These images are subsequently processed to extract deep features and mapped into granularity-specific embedding representations. Building on these embeddings, P2IFormer employs a multi-granularity self-attention mechanism to model both intra-granularity and inter-granularity contextual dependencies, achieving deep fusion of features across different granularities. Finally, a linear projection layer followed by a softmax function is used to perform fault classification. The experimental results under various constant-speed and variable-speed conditions demonstrate that the proposed method achieves excellent diagnostic performance, significantly outperforming existing CNN- and Transformer-based models. The main contributions of this paper are summarized as follows:

(1) A fault diagnosis model for high-speed train axle-box bearings, named P2IFormer, is developed based on a patch-to-image embedding framework. By combining multi-granularity segmentation of time series with image transformation, patch sequences at different granularities are converted into multi-channel Gramian Angular Field (GAF) images, significantly enhancing the modeling of local temporal features.
(2) A granularity-specific image embedding module is designed to generate feature representations for each granularity. Patch images at each granularity are processed through feature extraction, pooling, and linear projection, and then uniformly encoded as granularity-specific tokens. This provides high-quality representations to support effective multi-granularity interaction modeling.
(3) The proposed method is evaluated under various constant-speed and variable-speed operating conditions. The results demonstrate that P2IFormer achieves over 99.5% accuracy across all scenarios, significantly outperforming existing CNN- and Transformer-based methods.

## 2. Related Work

### 2.1. Transformer-Based Approaches for Bearing Fault Diagnosis

In recent years, the Transformer architecture has gained significant attention in the field of fault diagnosis due to its superior global modeling capability [26]. Compared with traditional convolutional neural networks (CNNs), Transformers leverage self-attention mechanisms to capture dependencies between arbitrary time steps, effectively addressing the limitations of CNNs, which are restricted to local feature extraction and struggle to model long-range temporal dependencies. In addition, Transformers support parallel computation and offer stronger representational capacity. In bearing fault diagnosis, some studies have applied or modified standard Transformer models to improve global feature extraction [27,28], while others have explored hybrid models that combine Transformers with CNNs to exploit their complementary strengths in local and global feature modeling [29,30]. However, most existing Transformer-based methods rely on fixed-length patch segmentation strategies, which hinder the ability to simultaneously capture multi-granularity temporal features and thus limit the modeling of complex fault patterns.

### 2.2. Image-Based Fault Diagnosis Through Time-Series Transformation

Transforming time-series data into images for fault diagnosis has emerged as an effective and rapidly evolving approach in recent years, with particularly notable success in bearing fault diagnosis. The core idea of this method is to map one-dimensional vibration signals into two-dimensional image representations using specific transformation algorithms, enabling powerful image-based models such as convolutional neural networks (CNNs) and vision Transformers to perform feature extraction and fault classification tasks [31]. Common transformation techniques include time-frequency representation methods such as short-time Fourier transform (STFT), wavelet transform (WT), and continuous wavelet transform (CWT), as well as structural mapping methods like Gramian Angular Field (GAF) and Markov Transition Field (MTF) [32,33,34,35,36]. These image-based techniques not only reveal the local dynamics and global trends within time-series data, but also enhance the ability of visual models to identify complex fault patterns.

### 2.3. Multi-Scale Feature Extraction for Fault Diagnosis

In recent years, multi-scale feature extraction has received increasing attention in bearing fault diagnosis, aiming to capture critical signal patterns distributed across different temporal and frequency ranges. The core idea behind such methods is to process vibration signals using varying receptive fields or segmentation granularities, thereby enabling joint modeling of both local details and global structures. In convolutional neural networks (CNNs), multi-scale architectures are typically constructed using parallel branches with different convolutional kernel sizes to extract and fuse features at multiple scales [37,38,39]. More recently, attention mechanisms have been incorporated into multi-scale CNN frameworks to enhance the model’s focus on salient features and improve the robustness of feature representations [40,41]. However, most existing multi-scale methods lack explicit modeling of the relationships between different scales, making it difficult to fully capture the semantic dependencies among features of varying scales.

## 3. Methodology

### 3.1. Problem Definition

The fault diagnosis of high-speed train axle-box bearings can be formulated as a multivariate time-series classification task. Let $x\in R^{T\times C}$ denote a segment of vibration signal, where *T* represents the sequence length (i.e., number of time steps) and *C* denotes the number of sensor channels (e.g., vibration, temperature). Each sample $x_{i}$ is associated with a label $y_{i}\in \left\{1,2,\ldots ,M\right\}$, indicating one of *M* predefined bearing conditions or fault types (e.g., normal, inner race fault, outer race fault). Given a training set ${\left\{\left(x_{i},y_{i}\right)\right\}}_{i=1}^{N}$ composed of *N* labeled instances, the objective is to learn a classification function $f:R^{T\times C}\to \left\{1,2,\ldots ,M\right\}$ that can accurately predict the fault category $\hat{y}=f\left(x\right)$ for any unseen sample *x*.

### 3.2. Overview of the P2IFormer Architecture

**Figure 2:**
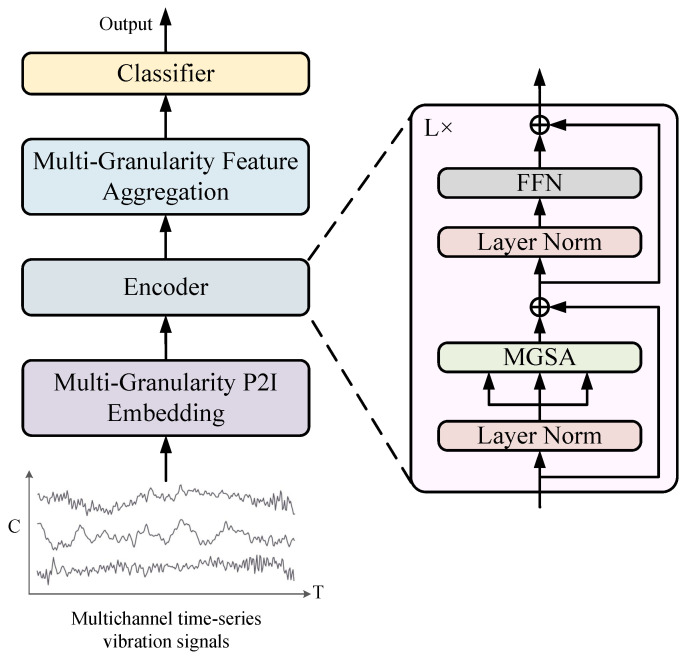
The overall architecture of the proposed P2IFormer model. The input consists of multi-channel vibration signal sequences collected from axle-box bearings. These signals are first processed by the Multi-Granularity P2I Embedding module, which splits the time series into patch sequences at multiple granularities and transforms them into image representations. The resulting embeddings are passed through a stack of encoder layers, each consisting of layer normalization, Multi-Granularity Self-Attention (MGSA), and a feed-forward network (FFN) with residual connections. The outputs from different granularities are integrated via the Multi-Granularity Information Aggregation module. Finally, the fused features are classified into fault categories using a softmax classifier.

The overall architecture of the proposed P2IFormer is illustrated in Figure 2. The input to the model is a multivariate vibration signal segment collected from axle-box bearings. First, the signal is processed by the Multi-Granularity Patch-to-Image (P2I) Embedding module, which partitions the sequence into multiple granularities using different window sizes and converts each patch into a Gramian Angular Field (GAF) image. Each image is then encoded into a latent token representation via a feature extraction module. Subsequently, the resulting multi-granularity embeddings are fed into a stack of encoder layers, each consisting of layer normalization, the Multi-Granularity Self-Attention (MGSA) module, and a feed-forward network. The MGSA mechanism models both intra-granularity and inter-granularity dependencies by leveraging learnable router tokens. To integrate the semantic information across different granularities, the updated router tokens from all granularities are concatenated in the Multi-Granularity Feature Aggregation module and projected into a unified feature space. Finally, the aggregated representation is passed through a fully connected layer followed by a softmax classifier to determine the bearing fault category.

### 3.3. Multi-Granularity Patch-to-Image Embedding

**Figure 3:**
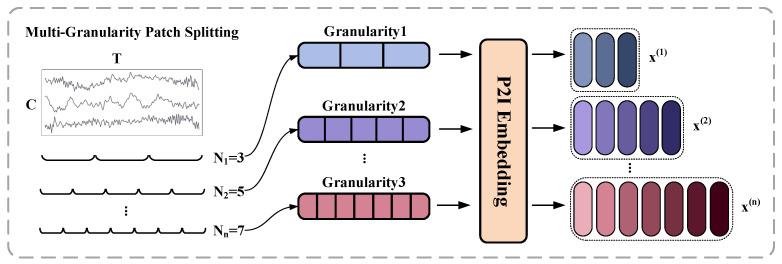
Illustration of the multi-granularity patch-to-image (P2I) embedding process.

To effectively capture temporal features across different scales, this study introduces a Multi-Granularity Patch-to-Image (P2I) Embedding module, as illustrated in Figure 3. The input multivariate time series is first divided into patch sequences under multiple granularity levels by applying sliding windows of different lengths. Each patch is then passed into the P2I module, which internally performs Gramian Angular Field (GAF) transformation and feature extraction through convolutional and attention-based operations. This process yields a token embedding for each patch. By applying the P2I module to all patches within each granularity level, a complete granularity-specific embedding sequence $x^{\left(i\right)}$ is constructed. The resulting embeddings across all granularities, denoted as $x^{\left(1\right)},x^{\left(2\right)},\ldots ,x^{\left(n\right)}$, serve as the foundation for subsequent multi-granularity attention modeling.

#### 3.3.1. Multi-Granularity Patch Splitting

To effectively capture fault-related patterns in axle-box bearing vibration signals across multiple temporal scales, this study introduces a multi-granularity sequence partitioning strategy. Let the original multivariate time series be $x\in R^{T\times C}$, where *T* denotes the sequence length and *C* is the number of sensor channels. A set of *n* patch lengths $\left\{l_{1},l_{2},\ldots ,l_{n}\right\}$ is selected. At granularity level *i*, the sequence *x* is divided into $N_{i}=T/l_{i}$ non-overlapping patches of length $l_{i}$, denoted as $P_{i,j}\in R^{l_{i}\times C},\;j=1,2,\ldots ,N_{i}$. This multi-granularity partitioning strategy enables the model to simultaneously capture fine-grained local dynamics (e.g., short-term transients) and coarse-grained global trends. It provides a rich and structured representation foundation for subsequent image transformation and deep feature extraction, thereby significantly enhancing the model’s temporal perception across multiple granularities in fault diagnosis.

#### 3.3.2. Patch-to-Image Embedding

**Figure 4:**
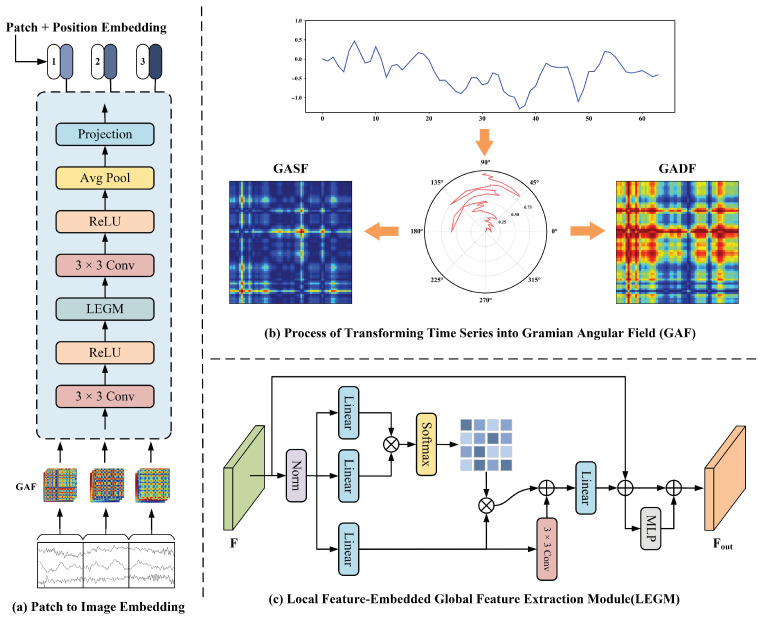
The architecture of the Patch-to-Image (P2I) Embedding module. Subfigure (**a**) illustrates the P2I embedding process at a granularity level where the time series is split into 3 non-overlapping patches, where each time-series patch is first transformed into a Gramian Angular Field (GAF) image. These images are then processed through a convolutional backbone combined with the Local Feature Embedded Global Feature Extraction Module (LEGM) module to extract deep semantic features. A projection layer finally outputs the embedding token for each patch. Subfigure (**b**) shows the GAF transformation procedure, encoding temporal dependencies via polar coordinate mapping. Subfigure (**c**) depicts the structure of the LEGM module, which jointly captures local spatial patterns and long-range dependencies through the integration of convolution and self-attention mechanisms.

To enhance the ability to learn local fault-sensitive features under multiple temporal scales, each patch at granularity level *i* is processed through the Patch-to-Image Embedding (P2I) module, as illustrated in Figure 4a. This module consists of three main stages: image transformation, deep feature extraction, and token generation. Ultimately, it produces a *d*-dimensional embedding vector for each patch.

First, each patch $P_{i,j}\in R^{l_{i}\times C}$, where $l_{i}$ denotes the patch length and *C* is the number of sensor channels, is converted into a 2D image using the Gramian Angular Field (GAF) technique [42], shown in Figure 4b. This method transforms the 1D time-domain subsequence into a 2D structured representation that captures temporal dependencies and dynamics. The GAF generation process includes the following steps:

- Step 1: Normalization. Each channel-wise sequence ${\left\{x_{k}\right\}}_{k=1}^{l_{i}}$ is normalized to the range [−1,1] using min–max scaling:
  (1) $${\tilde{x}}_{k}=\frac{2\left(x_{k}-\min_{k=1}^{l_{i}}x_{k}\right)}{\max_{k=1}^{l_{i}}x_{k}-\min_{k=1}^{l_{i}}x_{k}}-1$$
- Step 2: Polar coordinate mapping. Each normalized data point ${\tilde{x}}_{k}$ is mapped to a point in the polar coordinate system.
  (2) $$\phi_{k}=\arccos\left({\tilde{x}}_{k}\right),\;r_{k}=\frac{k}{l_{i}}$$
  where $\phi_{k}$ and $r_{k}$ represent the angular coordinate and radial coordinate in the polar space, respectively.
- Step 3: GAF image generation. The Gram Angular Summation Field (GASF) and Gram Angular Difference Field (GADF) are defined as
  (3) $${\mathrm{GASF}}_{i,j}=\cos\left(\phi_{i}+\phi_{j}\right),\;{\mathrm{GADF}}_{i,j}=\sin\left(\phi_{i}-\phi_{j}\right)$$
  where $i,j\in \{1,2,\ldots ,l_{i}\}$ denote the row and column indices in the resulting GAF image and $\phi_{i}$ and $\phi_{j}$ are the polar angles corresponding to the time-series values at time steps *i* and *j*, respectively.

Each channel undergoes the above process independently, and the resulting $l_{i}\times l_{i}$ GAF images are stacked along the channel dimension, forming a multi-channel image of shape $l_{i}\times l_{i}\times C$. To extract high-level semantic features, the image is first processed by a 3×3 convolution followed by a ReLU activation:

(4) $$F=\mathrm{ReLU}({\mathrm{Conv}}_{3\times 3}\left(\mathrm{GAF}\left(P_{i,j}\right)\right))$$

where *F* denotes the intermediate feature map.

Next, the Local Feature-Embedded Global Feature Extraction Module (LEGM), shown in Figure 4c, is applied to capture both fine-grained local details and long-range dependencies through a hybrid structure combining convolution and self-attention [43]. Given the feature map *F*, three learnable linear transformations are applied to produce the query *Q*, key *K*, and value *V*:

(5) $$Q=W_{q}F,\;K=W_{k}F,\;V=W_{v}F$$

where $W_{q},W_{k},W_{v}$ are learnable projection matrices. The attention map *A* and attention-enhanced feature $F_{a}ttn$ are then computed using scaled dot-product attention:

(6) $$A=\mathrm{softmax}\left(\frac{QK^{⊤}}{\sqrt{D}}\right),\;F_{\mathrm{att}}=AV$$

where *D* is the feature dimension. In addition, *V* is passed through a convolutional layer to emphasize spatial locality, producing $F_{c}onv$, which is fused with the attention output:

(7) $$F_{\mathrm{fuse}}=F_{\mathrm{att}}+F_{\mathrm{conv}}$$

The fused feature map is then passed through a linear layer and added residually to the original convolution-enhanced map, followed by an MLP layer:

(8) $$\hat{F}=\mathrm{Linear}\left(F_{\mathrm{fuse}}\right)+F,\;F_{\mathrm{out}}=\mathrm{MLP}\left(\hat{F}\right)+\hat{F}$$

To obtain the final token representation for the patch, $F_{o}ut$ is processed by convolution, ReLU activation, global average pooling, and a linear projection:

(9) $$e_{i,j}=W_{p}\cdot \mathrm{AvgPool}\left(\mathrm{ReLU}\left({\mathrm{Conv}}_{3\times 3}\left(F_{\mathrm{out}}\right)\right)\right)+b_{p},\;e_{i,j}\in R^{d}$$

where $W_{p}$ and $b_{p}$ are learnable parameters and $e_{i,j}$ is the patch-level token. To enhance the model’s sensitivity to patch ordering and temporal structure, a learnable positional encoding is added. Specifically, each patch position *j* at granularity level *i* is associated with a trainable positional vector ${\mathrm{pos}}_{i,j}\in R^{d}$, which is added to the patch embedding:

(10) $$z_{i,j}=e_{i,j}+{\mathrm{pos}}_{i,j},\;z_{i,j}\in R^{d}$$

where $z_{i,j}$ denotes the final token embedding for the *j*-th patch at granularity level *i*. Finally, the embedding vectors of all patches at granularity level *i* form a sequence-level embedding:

(11) $$x^{\left(i\right)}=\left\{z_{i,1},z_{i,2},\ldots ,z_{i,N_{i}}\right\}\in R^{N_{i}\times d}$$

where $N_{i}$ is the number of patches at granularity level *i* and *d* is the embedding dimension. After converting all patches into embeddings at their respective granularities, the original multivariate time series is represented as a set of granularity-specific embeddings.

### 3.4. Multi-Granularity Self Attention

To effectively model both intra- and inter-granularity contextual dependencies, a multi-granularity self-attention module is introduced. This module consists of two sequential stages: intra-granularity self-attention and inter-granularity self-attention [44]. First, for each of the *n* granularity levels, a learnable router vector $u^{\left(i\right)}\in R^{d}$ is initialized to summarize the global semantic representation at that granularity. These router vectors serve not only as semantic anchors within each granularity but also as bridges for communication across different granularities. For the *i*-th granularity level with $N_{i}$ patches, the router vector is initialized as the sum of a trainable global token embedding $e^{\left(i\right)}\in R^{d}$ and a learnable positional encoding ${\mathrm{pos}}_{N_{i}+1}^{\left(i\right)}\in R^{d}$ at the position $N_{i}+1$, formulated as

(12) $$u^{\left(i\right)}=e^{\left(i\right)}+{\mathrm{pos}}_{N_{i}+1}^{\left(i\right)},\;u^{\left(i\right)}\in R^{d}$$

#### 3.4.1. Intra-Granularity Self-Attention

At each granularity level *i*, the patch embedding sequence $x^{\left(i\right)}\in R^{N_{i}\times d}$ is concatenated vertically with its corresponding router vector $u^{\left(i\right)}\in R^{1\times d}$ to form the extended sequence

(13) $${\tilde{x}}^{\left(i\right)}=\left[x^{\left(i\right)}|u^{\left(i\right)}\right]\in R^{(N_{i}+1)\times d}$$

where the symbol [·∣·] denotes concatenation along the row dimension and the router vector is added as an additional token after the patch embeddings. Next, standard multi-head self-attention is applied:

(14) $$Q={\tilde{x}}^{\left(i\right)}W_{Q},\;K={\tilde{x}}^{\left(i\right)}W_{K},\;V={\tilde{x}}^{\left(i\right)}W_{V}$$

(15) $${\mathrm{Attn}}_{\mathrm{intra}}\left({\tilde{x}}^{\left(i\right)}\right)=\mathrm{softmax}\left(\frac{QK^{⊤}}{\sqrt{d}}\right)V\in R^{(N_{i}+1)\times d}$$

Here, $Q,K,V\in R^{(N_{i}+1)\times d}$ are the query, key, and value matrices and $W_{Q},W_{K},W_{V}$ are trainable linear projection weights. This intra-granularity attention focuses on learning temporal dependencies within each individual granularity level.

#### 3.4.2. Inter-Granularity Self-Attention

After intra-granularity self-attention is completed for all levels, the updated router vectors ${\hat{u}}^{\left(1\right)},{\hat{u}}^{\left(2\right)},\ldots ,{\hat{u}}^{\left(n\right)}$ each contain global semantic information representative of their respective granularity levels. These router vectors encode the contextual dependencies aggregated from the patch embeddings within their own granularities, effectively summarizing granularity-specific features. To enable cross-granularity interaction, all updated router vectors are concatenated to form the cross-granularity router matrix

(16) $$U=\left[{\hat{u}}^{\left(1\right)}\left|{\hat{u}}^{\left(2\right)}\right.\right|\ldots \left|{\hat{u}}^{\left(n\right)}\right]\in R^{n\times d}$$

Next, standard multi-head self-attention is applied over the router sequence to model inter-granularity semantic dependencies:

(17) $$Q^{\prime }=UW_{Q}^{\prime },\;K^{\prime }=UW_{K}^{\prime },\;V^{\prime }=UW_{V}^{\prime }$$

(18) $${\mathrm{Attn}}_{\mathrm{inter}}\left(U\right)=\mathrm{softmax}\left(\frac{Q^{\prime }{K^{\prime }}^{⊤}}{\sqrt{d}}\right)V^{\prime }\in R^{n\times d}$$

where $W_{Q}^{\prime },W_{K}^{\prime }$, and $W_{V}^{\prime }$ are learnable projection matrices.

### 3.5. Multi-Granularity Feature Aggregation

After processing through the encoder with multi-granularity self-attention, each updated router vector ${\hat{u}}^{\left(i\right)}\in R^{d}$ encodes both intra- and inter-granularity semantic information specific to granularity level *i*. To integrate global representations from all *n* granularities, we concatenate these router vectors along the feature dimension to form a single high-dimensional fused vector:

(19) $$u_{\mathrm{cat}}=\mathrm{concat}\left({\hat{u}}^{\left(1\right)},{\hat{u}}^{\left(2\right)},\ldots ,{\hat{u}}^{\left(n\right)}\right)\in R^{n\cdot d}$$

Here, concat(·) denotes the concatenation operation along the feature axis. Subsequently, the fused vector is projected to a target dimension $d^{\prime }$ via a fully connected layer followed by a GeLU activation function:

(20) $$h=\mathrm{GeLU}\left(W_{f}u_{\mathrm{cat}}+b_{f}\right),\;W_{f}\in R^{d^{\prime }\times \left(n\cdot d\right)},\;b_{f}\in R^{d^{\prime }}$$

where $W_{f}$ and $b_{f}$ are the learnable parameters of the projection layer. The output $h\in R^{d^{\prime }}$ serves as the final fused representation, integrating semantic features from all granularities. This information-rich vector is then used as the input to the classifier for fault type prediction.

### 3.6. Classifier

After multi-granularity feature aggregation, a unified representation vector $h\in R^{d^{\prime }}$ is obtained, encapsulating both global and cross-granularity semantic information. In the classification stage, this vector is fed into a simple yet effective discriminator to predict the fault category. Specifically, for a classification task with *M* fault types, the vector *h* is passed through a classifier composed of a fully connected layer followed by a softmax activation to produce the class probability distribution

(21) $$\hat{y}=\mathrm{Softmax}\left(\mathrm{Wh}+b\right)\in R^{M}$$

where $W\in R^{M\times d^{\prime }}$ and $b\in R^{M}$ are learnable parameters. The output $\hat{y}=\left[{\hat{y}}_{1},{\hat{y}}_{2},\ldots ,{\hat{y}}_{M}\right]$ represents the predicted probability for each fault class. During training, the model is optimized using the cross-entropy loss to measure the discrepancy between the predicted probability distribution $\hat{y}$ and and the ground truth label *y*.

## 4. Experiments and Results

### 4.1. Dataset Description

**Figure 5:**
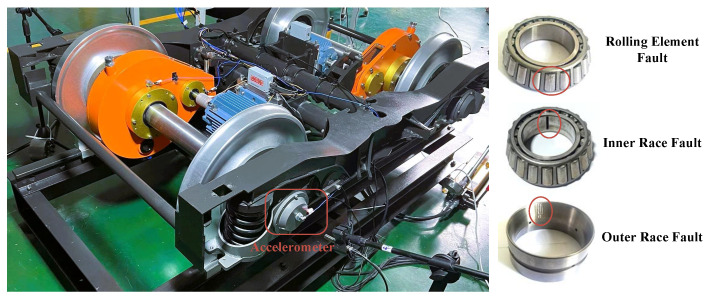
Axle-box bearing experimental platform.

The dataset used in this study was collected from an experimental platform simulating the axle-box bearings of high-speed trains, specifically using the HRB352213 bearing model. It includes one healthy state and four typical fault types: inner race fault, outer race fault, rolling element fault, and combination fault. Vibration signals were acquired using a triaxial accelerometer mounted on one side of the bearing, capturing three-channel data that comprehensively reflect the dynamic characteristics of the bearing. A photograph of the experimental platform is shown in Figure 5. During the experiments, the bearings were operated under four constant-speed conditions (20 Hz, 40 Hz, 60 Hz, and 80 Hz) and one variable-speed condition. In the variable-speed scenario, the rotation speed follows a triangular waveform: it increases linearly from 0 Hz to 40 Hz and then decreases linearly back to 0 Hz, with the entire cycle lasting approximately 2 s and peaking at 1 s. This speed variation simulates the actual acceleration and deceleration processes of high-speed trains, enabling the evaluation of the model’s robustness under dynamic operating conditions. The vibration signals were sampled at a frequency of 25.6 kHz, and each sample has a duration of 10 s, ensuring sufficient resolution for capturing fault-related features.

### 4.2. Experimental Setup

**Figure 6:**
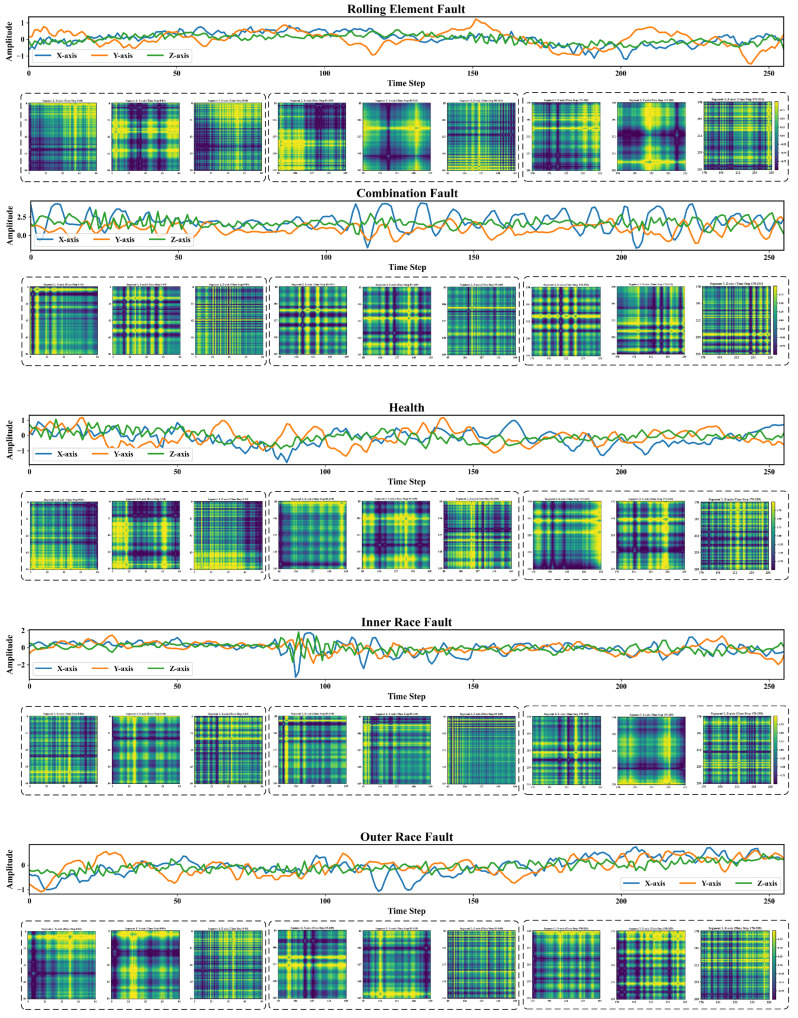
Visualization of vibration signal segments for each fault category under the 80 Hz rotational speed condition and their corresponding GADF images (patch length = 85).

In this study, the collected vibration signals were segmented into training samples using a sliding window technique with a window size of 256 and a step size of 64. As a result, 4093 samples were generated for each fault category, yielding a total of 20,465 samples for model training, validation, and testing. Each vibration signal sample was segmented into three granularity levels using predefined patch lengths of 85, 51, and 36, respectively. At each level, the sample was divided into non-overlapping patches of the corresponding length. These patches were subsequently transformed into Gramian Angular Field (GAF) images, with the resulting image size equal to the respective patch length (i.e., 85 × 85, 51 × 51, and 36 × 36 for each granularity level). Figure 6 illustrates example vibration signal samples for each fault category at the 80 Hz rotational speed, along with their corresponding GADF images at the patch length of 85. The entire dataset was divided into training, validation, and test sets in a 60%:20%:20% ratio.

The overall performance and stability of the proposed model are influenced by various architectural and hyperparameter settings, such as the embedding dimension and the number of attention heads. The detailed network configuration and hyperparameter values adopted in this study are summarized in Table 1.

To comprehensively evaluate the robustness and generalization capability of the proposed P2IFormer model under varying operating conditions, extensive experiments were conducted across four constant-speed scenarios (20 Hz, 40 Hz, 60 Hz, and 80 Hz), as well as one variable-speed condition. Several representative baseline models were selected for comparison, including CNN-LSTM [45], WDCNN [46], DenseNet [36], ResNet [47], Vision Transformer (ViT) [48], and ECMCTP [24]. All experiments were implemented using the PyTorch 2.0 deep learning framework in a Python 3.8 environment and executed on a workstation equipped with an NVIDIA RTX 3090 GPU (24 GB memory).

To quantitatively assess the performance of the models, average accuracy and F1-score were employed as evaluation metrics. Their definitions are as follows:

(22) $$\mathrm{Accuracy}=\frac{1}{M}\sum_{i=1}^{M}\frac{{\mathrm{TP}}_{i}+{\mathrm{TN}}_{i}}{{\mathrm{TP}}_{i}+{\mathrm{TN}}_{i}+{\mathrm{FP}}_{i}+{\mathrm{FN}}_{i}}$$

(23) $${F1}_{i}=2\times \frac{{\mathrm{Precision}}_{i}\times {\mathrm{Recall}}_{i}}{{\mathrm{Precision}}_{i}+{\mathrm{Recall}}_{i}}$$

(24) $${\mathrm{Precision}}_{i}=\frac{{\mathrm{TP}}_{i}}{{\mathrm{TP}}_{i}+{\mathrm{FP}}_{i}},\;{\mathrm{Recall}}_{i}=\frac{{\mathrm{TP}}_{i}}{{\mathrm{TP}}_{i}+{\mathrm{FN}}_{i}}$$

Here, ${\mathrm{TP}}_{i}$, ${\mathrm{TN}}_{i}$, ${\mathrm{FP}}_{i}$, and ${\mathrm{FN}}_{i}$ denote the true positives, true negatives, false positives, and false negatives for class *i*, respectively. These metrics effectively reflect classification performance and model generalization, particularly under class-imbalanced multi-class conditions.

### 4.3. Performance Evaluation Under Constant-Speed Conditions

**Figure 7:**
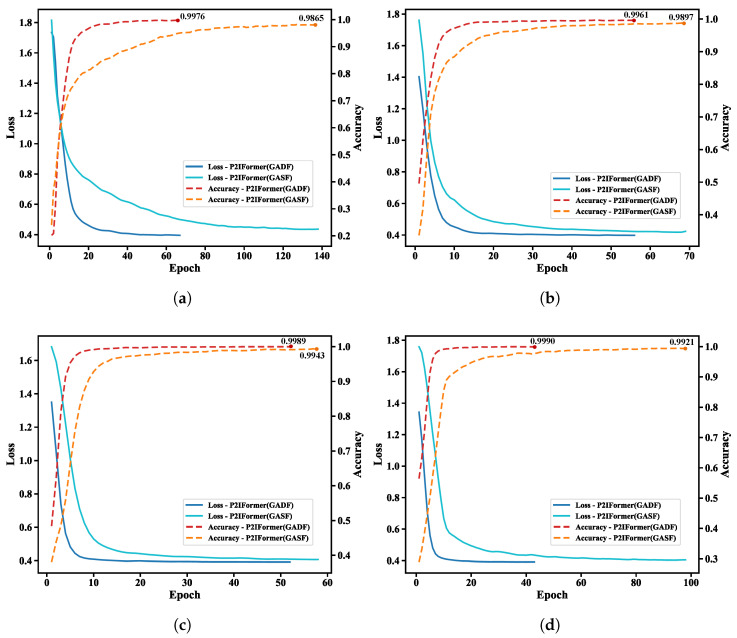
Validation loss and accuracy curves of the P2IFormer model under four constant-speed conditions using GASF and GADF encodings: (**a**) 20 Hz, (**b**) 40 Hz, (**c**) 60 Hz, and (**d**) 80 Hz.

To evaluate the fault recognition capability of the proposed P2IFormer model under varying operating conditions, the model was independently trained and validated under four constant-speed scenarios: 20 Hz, 40 Hz, 60 Hz, and 80 Hz. During the patch-to-GAF image transformation stage, two Gramian Angular Field encoding methods—Gramian Angular Summation Field (GASF) and Gramian Angular Difference Field (GADF)—were employed to examine their effects on model performance. The corresponding loss and accuracy curves on the validation set are illustrated in Figure 7.

As shown in the Figure 7, P2IFormer with GADF encoding consistently exhibits faster convergence and higher final accuracy across all speed conditions. For instance, at 20 Hz (Figure 7a) and 40 Hz (Figure 7b), the GADF-based model achieves rapid convergence within the first 20 epochs and reaches final accuracies of 99.76% and 99.61%, respectively, while the GASF-based model lags slightly behind, with accuracies of 98.65% and 98.97%. Similarly, under the 60 Hz (Figure 7c) and 80 Hz (Figure 7d) conditions, the GADF-based model maintains superior performance, achieving over 99.8% accuracy and demonstrating excellent robustness and stability.

These results confirm that GADF encoding more effectively preserves local temporal variations and enhances the discriminative representation of fault patterns, enabling P2IFormer to achieve consistently high diagnostic accuracy across varying rotational speeds. The superiority of GADF over GASF lies in its distinct mathematical formulation and its capacity to capture critical temporal dynamics. Specifically, the GADF matrix is constructed based on angular differences ($\phi_{i}-\phi_{j}$), resulting in an anti-symmetric structure ($GADF_{i,j}=-GADF_{j,i}$) that encodes directional information and intuitively reflects upward or downward trends within the sequence. Moreover, its diagonal elements are always zero, eliminating redundant self-correlations and yielding a more compact and efficient representation. In contrast, GASF relies on angular summation ($\phi_{i}+\phi_{j}$), producing a symmetric matrix ($GASF_{i,j}=GASF_{j,i}$) that lacks directional sensitivity. Additionally, its non-zero diagonal elements retain self-correlations, which may introduce irrelevant redundancy in classification tasks. Consequently, GADF facilitates faster model convergence and improves classification performance during training. Therefore, GADF was selected as the default encoding method for subsequent comparative experiments against baseline models such as DenseNet, ResNet, ViT, and the proposed P2IFormer. The final evaluation results are summarized in Table 2.

As shown in Table 2, the proposed P2IFormer achieves the highest accuracy and F1-score under the 20 Hz, 40 Hz, and 60 Hz speed conditions, demonstrating superior fault classification performance. At the 80 Hz high-speed condition, the DenseNet model slightly outperforms P2IFormer, yielding the best results. Overall, the performance advantage of P2IFormer becomes more evident as the rotational speed decreases, indicating stronger robustness and generalization capability under low-speed scenarios.

**Figure 8:**
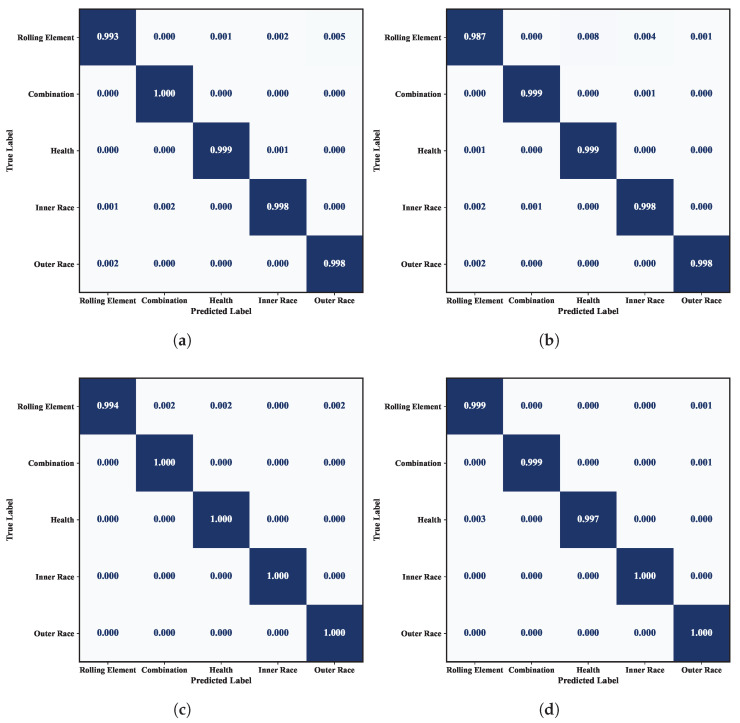
Confusion matrices of the P2IFormer model under constant-speed conditions: (**a**) 20 Hz, (**b**) 40 Hz, (**c**) 60 Hz, and (**d**) 80 Hz.

In terms of model complexity, P2IFormer contains 17.14 million parameters, which is significantly higher than lightweight models such as WDCNN (0.029 M), CNN-LSTM (0.093 M), and ECMCTP (0.781 M), but remains much smaller than large-scale models like ResNet (42.51 M) and ViT (85.80 M). Although the training time per epoch (43 s) is moderately higher compared to lightweight models, P2IFormer maintains a reasonable computational cost, making it feasible for deployment in environments with sufficient computational resources. The confusion matrices under different speed conditions, as shown in Figure 8, further validate the stability and effectiveness of P2IFormer in multi-class fault diagnosis tasks.

### 4.4. Performance Evaluation Under the Variable-Speed Condition

**Figure 9:**
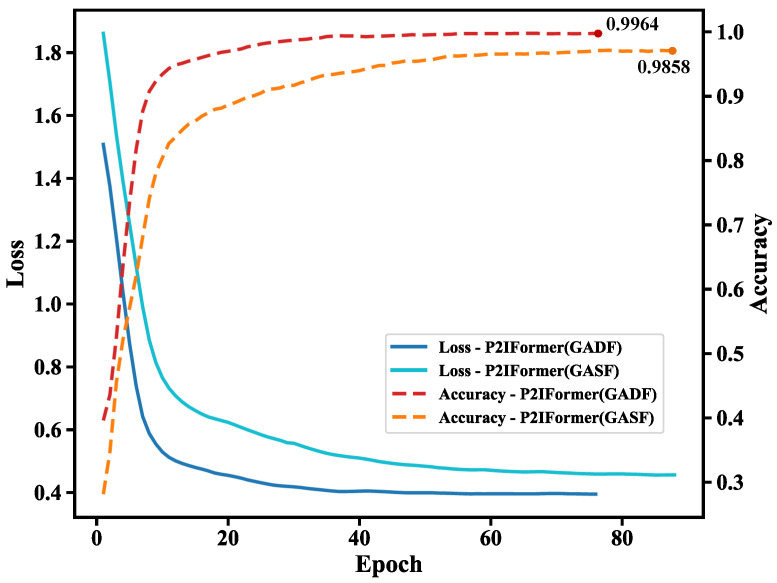
Validation loss and accuracy curves of P2IFormer using GASF and GADF encodings under the variable-speed condition.

Similar to the experiments conducted under constant-speed conditions, the performance of the proposed model was further evaluated under a variable-speed scenario. As shown in Figure 9, the validation loss and accuracy curves demonstrate the model’s effectiveness in handling non-stationary signals caused by speed variations. Compared to constant-speed settings, variable-speed conditions better reflect real-world operating environments, posing greater challenges to the model’s robustness and generalization capability. The results show that the model maintains strong convergence and diagnostic performance under these complex conditions. Notably, when using the GADF encoding, the model achieves faster convergence and higher accuracy, reaching 99.64% on the validation set, compared to 98.58% with GASF encoding.

**Figure 10:**
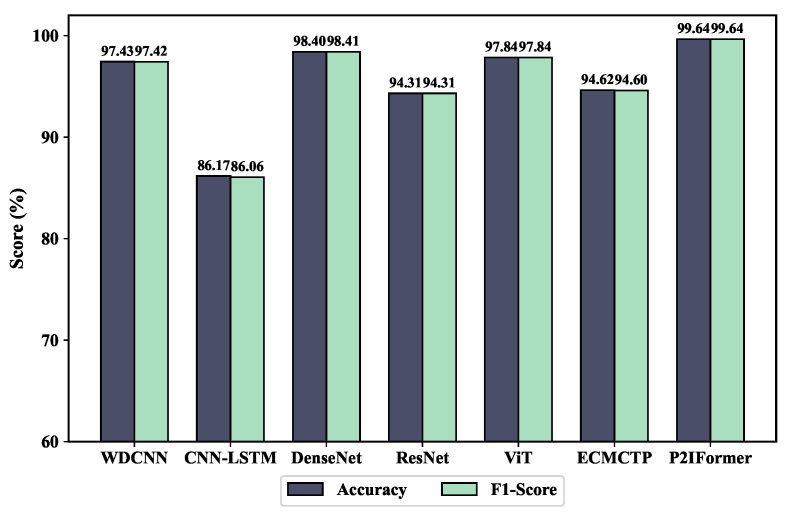
Comparison of accuracy and F1-score for different models under variable-speed conditions.

**Figure 11:**
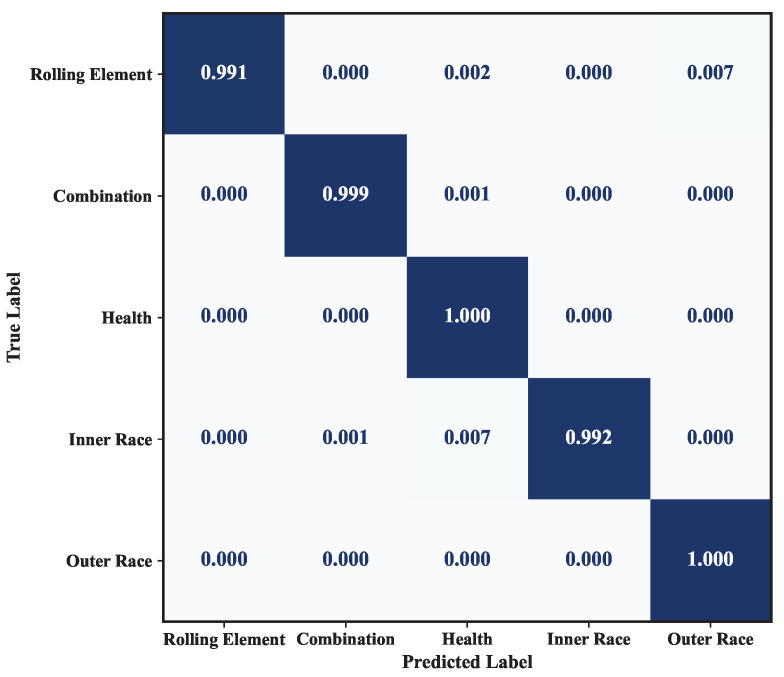
Confusion matrix of the proposed P2IFormer under the variable-speed condition.

The fault diagnosis performance of different models under the variable-speed condition is compared in Figure 10. As shown, the CNN-LSTM model performs the worst, achieving only 86.17% accuracy and an F1-score of 86.06%. This indicates its limited robustness in handling the the non-stationary characteristics of vibration signals caused by varying speed. In contrast, the proposed P2IFormer achieves the best performance, with both accuracy and F1-score reaching 99.64%. This demonstrates its superior capability in modeling temporal dependencies and integrating multi-granularity features, making it more adaptive to complex operating conditions. ResNet, ViT, and DenseNet also achieve relatively high accuracy and F1-scores. Among them, DenseNet reaches 98.40% accuracy, slightly outperforming the other two models, though still exhibiting a performance gap compared to P2IFormer. WDCNN performs moderately well but falls short of ResNet and ViT, particularly in handling non-stationary input sequences. The confusion matrix results on the test set are illustrated in Figure 11.

### 4.5. Ablation Study

**Figure 12:**
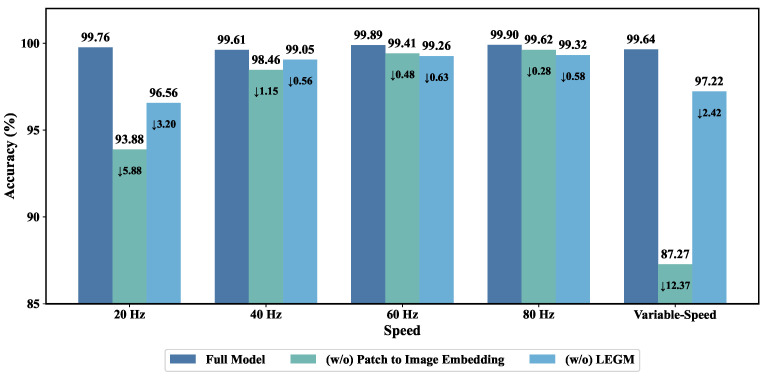
Ablation study results on the effectiveness of the patch-to-image embedding and LEGM.

To evaluate the contribution of each core component in the proposed P2IFormer model, a series of ablation experiments were conducted. The corresponding results are illustrated in Figure 12 and Table 3.

As shown in Figure 12, to validate the effectiveness of the Patch-to-Image Embedding module, we replaced it with a traditional patch embedding method, where each patch is directly projected into a high-dimensional vector using a multilayer perceptron (MLP). The results show that this substitution leads to a significant drop in accuracy under all speed conditions, with reductions of 5.88% and 12.37% observed in the 20 Hz and variable-speed scenarios, respectively. This demonstrates the advantage of image-based embedding in capturing spatiotemporal structures from raw sequences. Additionally, we investigated the impact of the Local Feature-Embedded Global Feature Extraction Module (LEGM) by removing it while preserving the patch-to-image embedding framework. In this setting, standard convolutional layers were used for feature extraction. Performance declined in all cases, indicating that LEGM contributes meaningfully by integrating local and global information.

To evaluate the effectiveness of the multi-granularity strategy, we designed three single-granularity variants with the number of patches per sample set to 3, 5, and 7, respectively. These models retain the core architecture but use standard self-attention for feature learning. As shown in Table 3, all single-granularity models underperform the full model, especially under the variable-speed and low-speed (20 Hz) settings. This demonstrates that the multi-granularity approach enhances the model’s ability to capture diverse temporal patterns and improves robustness under non-stationary conditions.

### 4.6. Robustness Evaluation Under Noisy Conditions

To further validate the robustness and practical applicability of the proposed model in real-world industrial environments, we conducted a comparative analysis of fault diagnosis performance across multiple baseline models under different noise levels. Specifically, Gaussian white noise with varying intensities was added to the original vibration signals to simulate five distinct signal-to-noise ratio (SNR) conditions: −6 dB, −3 dB, 0 dB, 3 dB, and 6 dB. This setup aims to replicate the typical background interference encountered in real applications. The SNR is defined as

(25) $${\mathrm{SNR}}_{\mathrm{dB}}=10\log_{10}\left(\frac{P_{\mathrm{signal}}}{P_{\mathrm{noise}}}\right)$$

where $P_{\mathrm{signal}}$ and $P_{\mathrm{noise}}$ represent the power of the signal and noise, respectively.

Under each SNR condition, the classification accuracy of all models was evaluated across five speed scenarios: 20 Hz, 40 Hz, 60 Hz, 80 Hz, and variable speed. As shown in Table 4, the proposed P2IFormer consistently achieved superior performance across most speed and noise settings, demonstrating excellent robustness against noise. Notably, under the low-speed (20 Hz) and variable-speed conditions, P2IFormer outperformed all other models by a significant margin, achieving 90.26% and 90.23% accuracy at −6 dB, respectively. Under the 80 Hz condition, ECMCTP achieves the highest accuracy across all noise levels. The DenseNet-based model also shows relatively stable performance across different conditions, ranking second only to P2IFormer in overall average accuracy.

### 4.7. Discussion

The experimental results indicate that, except for the 80 Hz condition where the DenseNet-based model with GADF encoding achieved comparable performance, P2IFormer consistently outperforms all baseline models in terms of accuracy and F1-score under the remaining constant-speed conditions, demonstrating remarkable stability and strong discriminative capability. Notably, its performance advantage is more pronounced under the low-speed (20 Hz) and variable-speed scenarios. This superiority is primarily attributed to the proposed innovative model architecture. On the one hand, the multi-granularity patching strategy, combined with time-series-to-image conversion using GAF encoding, enables the model to capture both local dynamics and global trends across multiple temporal granularities, thereby enriching the diversity and expressiveness of feature representations. On the other hand, the incorporation of a multi-granularity self-attention mechanism effectively models both intra- and inter-granularity contextual dependencies, further enhancing the integration of semantic information.

The ablation study confirms that the Patch-to-Image Embedding module, Local Feature-Embedded Global Feature Extraction Module (LEGM), and the multi-granularity strategy all contribute positively to the final performance, validating the rationality of the proposed architectural design. Furthermore, in the robustness evaluation under varying noise levels (SNR = −6 dB to 6 dB), P2IFormer consistently maintained high diagnostic accuracy across all rotational speeds. Its performance remained stable even under low-SNR and variable-speed conditions, highlighting its strong noise resistance and adaptability to complex environments.

In terms of model complexity and computational cost, P2IFormer comprises 17.14 million parameters, which is substantially lower than ViT (85.80 M) and ResNet (42.51 M), but significantly higher than lightweight models such as WDCNN (0.029 M), CNN-LSTM (0.093 M), and ECMCTP (0.781 M). Moreover, P2IFormer also incurs a higher per-epoch training time compared to these lightweight baselines, indicating a greater demand for computational resources.

## 5. Conclusions

This paper proposes a deep learning model named P2IFormer, which is designed to address the limitations of existing fault diagnosis methods for high-speed train axle-box bearings in multi-scale modeling and temporal feature extraction. The proposed model first segments the raw vibration signals using multiple window lengths, generating patch sequences at various granularities to enable structured modeling across different temporal scales. Each patch is then transformed into a corresponding Gramian Angular Field (GAF) image, enhancing the representation of local temporal features. A Local Feature-Embedded Global Feature Extraction module is subsequently applied to extract deep semantic features and generate token embedding at each granularity. Furthermore, a multi-granularity self-attention mechanism is employed to capture both intra- and inter-granularity semantic dependencies, effectively integrating fault information across granularities and improving the model’s capability to distinguish complex fault patterns. Experimental results demonstrate that P2IFormer achieves fault diagnosis accuracy exceeding 99.5% under four constant-speed conditions and one variable-speed condition, significantly outperforming mainstream Transformer- and CNN-based models. These results validate the model’s strong robustness and generalization capability across diverse operating scenarios. Furthermore, ablation and noise robustness experiments confirm the effectiveness of the model’s key components and its resilience to noise interference. Nevertheless, P2IFormer exhibits a larger parameter size and longer training time compared to lightweight models such as WDCNN and ECMCTP, indicating higher computational requirements. Future research will focus on lightweight model design to reduce computational resource requirements and improve deployment efficiency. Additionally, efforts will be directed toward developing improved strategies for handling scenarios with limited fault samples and imbalanced datasets, aiming to enhance the model’s adaptability and robustness in real-world industrial applications.

## Figures and Tables

**Table 1:** Hyperparameter settings of the proposed P2IFormer model.

Hyperparameter	Value
Input sequence shape	256×3
Number of granularities *n*	3
Patch lengths $\left\{l_{1},l_{2},l_{3}\right\}$	{85,51,36}
Embedding dimension *d*	128
Encoder layers *L*	2
Attention heads *H*	4
Dropout rate	0.2
Learning rate	$1\times 10^{-4}$
Batch size	64
Fusion vector dimension $d^{\prime }$	768
Loss function	Cross-Entropy
Optimizer	AdamW
Weight decay	$1\times 10^{-4}$

**Table 2:** Comparison of fault diagnosis performance across different models under four constant-speed conditions. Accuracy and F1-score are expressed as percentages (%).

Model	20 Hz		40 Hz		60 Hz		80 Hz		Params	Time/Epoch
	**Acc**	**F1**	**Acc**	**F1**	**Acc**	**F1**	**Acc**	**F1**	**(M)**	**(s)**
WDCNN	91.29	90.94	98.78	98.77	99.45	99.45	99.56	99.56	0.029	3
CNN-LSTM	90.99	90.95	97.51	97.50	99.27	99.27	99.67	99.67	0.093	5
DenseNet	97.84	97.82	99.29	99.28	99.80	99.80	**99.98**	**99.98**	6.959	81
ResNet	92.93	92.90	99.39	99.38	99.28	99.28	99.40	99.40	42.510	66
ViT	95.12	95.10	99.12	99.11	99.42	99.42	99.38	99.38	85.801	140
ECMCTP	96.38	96.36	99.41	99.40	99.52	99.52	99.96	99.96	0.781	25
**P2IFormer**	**99.76**	**99.76**	**99.61**	**99.61**	**99.89**	**99.89**	99.90	99.90	17.143	43

**Table 3:** Performance of single-granularity variants and accuracy drop compared to the full multi-granularity model.

Model	20 Hz	40 Hz	60 Hz	80 Hz	Variable Speed
**P2I-MG (multi)**	**99.76**	**99.61**	**99.89**	**99.90**	**99.64**
P2I-3 (single)	96.93	98.70	98.88	98.90	97.41
	↓ *2.83*	↓ *0.91*	↓ *1.01*	↓ *1.00*	↓ *2.23*
P2I-5 (single)	96.87	98.39	99.02	99.09	97.87
	↓ *2.89*	↓ *1.22*	↓ *0.87*	↓ *0.81*	↓ *1.77*
P2I-7 (single)	97.48	98.25	99.04	99.18	97.23
	↓ *2.28*	↓ *1.36*	↓ *0.85*	↓ *0.72*	↓ *2.41*

**Table 4:** Classification accuracy (%) under different SNR levels grouped by noise level.

SNR (dB)	Model	20 Hz	40 Hz	60 Hz	80 Hz	Variable-Speed
−6	WDCNN	57.46	63.75	77.19	76.44	50.35
	CNN-LSTM	60.12	65.20	80.19	79.88	51.62
	DenseNet	88.24	94.15	98.53	99.43	87.41
	ResNet	86.51	94.59	94.40	98.45	86.98
	ViT	77.18	90.14	91.69	92.05	76.93
	ECMCTP	78.97	92.39	98.47	**99.61**	79.52
	P2IFormer	**90.26**	**97.46**	**98.58**	97.76	**90.23**
−3	WDCNN	58.75	65.82	78.09	79.10	52.50
	CNN-LSTM	62.38	71.19	83.27	85.97	55.23
	DenseNet	90.28	**97.70**	98.70	98.57	88.70
	ResNet	89.02	96.35	90.29	98.39	88.79
	ViT	79.35	92.93	93.47	94.02	82.60
	ECMCTP	81.98	92.85	97.87	**99.69**	81.10
	P2IFormer	**91.51**	97.26	**98.80**	97.73	**91.91**
0	WDCNN	61.80	75.27	87.21	87.50	59.86
	CNN-LSTM	65.71	78.74	88.28	91.04	61.47
	DenseNet	90.89	98.57	**99.10**	99.21	91.77
	ResNet	90.29	96.19	98.03	98.75	90.45
	ViT	82.26	94.61	94.28	96.29	84.28
	ECMCTP	84.18	94.20	99.06	**99.90**	84.57
	P2IFormer	**92.64**	**98.78**	**99.10**	98.60	**93.71**
3	WDCNN	69.14	84.91	92.36	94.80	65.82
	CNN-LSTM	70.54	86.62	91.62	95.79	65.12
	DenseNet	91.81	98.78	**99.32**	99.36	93.27
	ResNet	90.79	98.06	99.02	99.09	92.90
	ViT	83.48	95.65	97.00	97.07	85.94
	ECMCTP	85.08	96.07	98.99	**99.93**	85.60
	P2IFormer	**93.91**	**99.06**	99.10	99.15	**95.32**
6	WDCNN	75.22	87.05	96.56	97.93	72.34
	CNN-LSTM	76.91	91.17	96.77	98.72	73.70
	DenseNet	94.07	99.10	**99.84**	99.65	94.20
	ResNet	91.73	**99.30**	99.24	99.25	93.16
	ViT	84.70	96.87	97.88	97.95	87.88
	ECMCTP	89.41	97.18	99.27	**99.95**	88.81
	P2IFormer	**95.51**	99.28	99.32	99.23	**95.48**

## Data Availability

The raw data supporting the conclusions of this article will be made available by the authors upon request.
